# Tracking and its Potential for Older Adults with Memory Concerns

**DOI:** 10.1145/3706598.3714093

**Published:** 2025

**Authors:** Amelia Short, Norman Makoto Su, Ruipu Hu, Eun Kyoung Choe, Hernisa Kacorri, Margaret Danilovich, David E. Conroy, Shannon Jette, Beth Barnett, Amanda Lazar

**Affiliations:** University of Maryland, College Park, Maryland, USA; Department of Computational Media, University of California, Santa Cruz, Santa Cruz, California, USA; College of Information, University of Maryland, College Park, Maryland, USA; College of Information, University of Maryland, College Park, Maryland, USA; College of Information, University of Maryland, College Park, Maryland, USA; CJE SeniorLife, Chicago, Illinois, USA; Kinesiology, The Pennsylvania State University, University Park, Pennsylvania, USA; University of Maryland, College Park, Maryland, USA; College of Information, University of Maryland, College Park, Maryland, USA; College of Information, University of Maryland, College Park, Maryland, USA

**Keywords:** Older adults, cognitive impairment, dementia, memory concerns, affect, stigma, tracking, self-tracking, ambiguity, Human-centered computing, Human computer interaction (HCI)

## Abstract

Much research on older people with memory concerns is focused on tracking and informed by the priorities of others. In this paper, we seek to understand the potential that people with memory concerns see in tracking. We conducted interviews with 29 participants with concerns about their memory and engaged in an affective writing approach. We find a range of potentials that can be traced to how participants are already self-tracking. Emotions associated with these potentials vary: from acceptance to resistance, and positive anticipation to aversion. Participants are emotionally motivated to foreclose possibilities in some instances and keep them open in others. While individual and unique, potential is structured by forces that include individual routines, relationships with others, and macro-level institutions and cultural contexts. We reflect on these findings in the context of research on self-tracking with older adults, designing with ambiguity, and forces that structure the experience of living with memory concerns.

## 1 Introduction

Set against the backdrop of the growing number of older people with cognitive impairment worldwide, technology researchers are working on initiatives to track people, symptoms, and outcomes. Tracking research includes innovative technical approaches to monitor cognitive scores [45, 48], detect events such as becoming lost [20, 81], and register people with age-related memory concerns in large databases [47]. Through these tracking initiatives, researchers are working towards futures where cognitive impairment is detected earlier [45, 48], threats to well-being are minimized [20, 81], and treatment breakthroughs can happen [47]. While these efforts are intended to support the health of people living with memory concerns, like most technology efforts for this population, they are often designed or framed for *others* to offer care [52, 65, 84]. Therefore, the current state of tracking for people with memory concerns may not be based on the actual health-related concerns, feelings, or priorities that exist related to tracking for this group, nor the possibilities they envision for their future [4]. Something else in common in these tracking initiatives is that they rarely involve the person with cognitive impairment in an agentic role – both in terms of data collection (i.e., passive sensing) as well as in who gets to access and make decisions about how to use the data [84].

*Self-tracking* (also known as self-monitoring [46], lifelogging [32], quantified self [15], or personal informatics [28]) is a form of tracking where an individual keeps track of their own behaviors, thoughts, and feelings. With technological advancements, self-tracking is often supported by Personal Informatics systems [27]. However, it has long been practiced manually, using pen and paper or simply relying on memory. Self-tracking inherently positions individuals in agentic roles, warranting them to make decisions about tool preparation, data collection, data integration, reflection, and action [51]. Therefore, studying self-tracking has the potential to offer alternative visions of tracking for people with cognitive concerns. In this paper, we study how a group of people above the age of 60 with memory concerns are already self-tracking, and the potential that they see in self-tracking. We interviewed 29 participants with a range of concerns about their memory. We asked participants questions about how they engaged in self-tracking to support their goals. We scoped our study to health-related tracking, with health defined broadly and including cognitive and emotional health in addition to physical health.

Research has found that tracking can be emotionally charged for older people [9, 12, 23, 80], particularly in the context of impaired cognition [9, 25]. We draw on past HCI research utilizing an analytic approach [77] inspired by Kathleen Stewart’s affective writing [73], which can surface situated emotions. This approach entails developing scenes from individual participants’ data and then arranging these scenes loosely [77]. The goal is to provide the reader (and writer) with “a way to experience the affect of ‘data’ in its rawness – to exude the nervous, charged, and happenstance trajectories of affect” (pp. 6–7, [77]).

Applying this analytic approach to our data led us to focus on *potential* — perceptions and emotions regarding what futures are possible for oneself — associated with self-tracking. Stewart’s work is consumed by potential. She describes how analytic approaches can assume states, such as “feeling worried,” to be fixed and inevitable results of causes that can be traced back in time [75]. For Stewart, a particular state is not inevitable, but involves “precise actualizations of a field of potentiality” (p. 519, [74]). Our focus in this paper is surfacing this “field of potentiality,” or what futures participants consider possible as a result of the ways they are currently tracking. The first two research questions, then, are: **What potentials can be traced to the ways that older people with memory concerns are already engaging in self-tracking (RQ1)? What emotions become visible as people consider these potentials (RQ2)?**

While each scene in this paper is unique, Stewart’s perspective is that potential, and people’s feelings about it, do not exist apart from broader structural forces [75]. Material, social, and political forces structure current experiences as well as future potentials [75]. For example, the socialization of what things one should be worried about, a body’s well-worn chemical pathways, and one’s position of power relative to a particular situation all affect whether we might feel worried in response to a particular piece of news about our health. These forces would also be involved in shaping the corresponding potentials, i.e., our perceptions and emotions regarding what futures are now possible. With this understanding of the relationship between structural forces and potential, our third research question is: **What are some of the forces structuring self-tracking-related potentials for older people with memory concerns (RQ3)?**

Our analysis reveals a range of potentials associated with self-tracking. Self-tracking is already underway, occurring naturally across widely varying domains in the lives of participants. Potentials include things being in the right place, losing weight, and improving one’s cognition, among others. Emotions range widely as well, including, for example, acceptance, resistance, unsettledness, and intrigue. We also find that some participants want tracking to foreclose possibilities. Others prefer to keep possibilities open.

As initiatives that seek to track people with memory concerns, their symptoms, and outcomes continue to rapidly develop and grow in scale, this paper contributes a slowed-down look at self-tracking in the context of everyday life with memory concerns. Our contributions are threefold. First, in a research landscape where people with cognitive concerns are rarely considered as potential users of the technologies that are designed for them [52, 65, 84], our study yields insights into sensitively approaching designing self-tracking technologies for this group, connecting with prior work on self-tracking with older adults [9, 22, 64, 79, 82]. Second, we contribute an understanding of how participants desire to keep some potentials open and foreclose others and the role of emotions and forms of tracking in these decisions. This opens new opportunities for design given that current tracking initiatives largely seek to resolve ambiguity. Finally, we contribute insights on how self-tracking with cognitive concerns can be understood in the context of structural forces, such as the cultural meanings which engender a phenomenon referred to as “dementia worry” [42].

## 2 Related Work

Below, we review research on older adults and self-tracking and the ways that emotions are involved in self-tracking.

### 2.1 Older adults and self-tracking

In this paper, we use a broad definition of self-tracking, which involves an individual keeping track of their own behaviors, thoughts, or feelings [46]. As Lupton puts it, self-tracking is an active and purposeful data practice that not only produces data assemblages but also transcends the collection and interpretation of information, serving as both self-narratives and performances of selfhood [56]. This multifaceted nature of self-tracking is reflected in the wide range of methods that can support it, including automated, manual, and hybrid approaches [14]. Furthermore, objects that evoke embodied feelings – such as an old pair of jeans providing a sense of one’s own body weight or size, or pencil marks on door jambs for tracking heights – along with noting these observations in one’s own head, are also considered forms of self-tracking [29, 56]. Self-tracking when defined as such is a very common health activity [29]. One 2013 report found that 71 percent of US adults over the age of 65 were self-tracking their weight, diet, or exercise routines through mechanisms including paper, mobile devices, medical devices, and in their heads [29]. Older adults often rely on memory instead of external tools for recording data, due to effort, routine disruption, tool-related challenges, avoidance of illness reminders, and fear of data loss [59].

Primarily focused on self-tracking with *technology*, researchers in HCI are unpacking factors that contribute to uptake and use and finding ways to make self-tracking technologies more useful and accessible for older adults. Most self-tracking research with older adults has focused on health [10, 11], which is not surprising given that self-tracking is of interest for supporting health for many populations. Studies have revealed findings that challenge taken-for-granted assumptions about designing good self-tracking systems for younger people. For example, one study found that older adults wanted to track health metrics (e.g., rest) in ways that were not (at that time) possible with commercially available apps, concluding that older adults should be involved throughout the technology design process [22]. Another study noted that tracking did not necessarily provide motivation to older participants to work towards health goals [9]. While self-tracking devices and apps typically provide feedback on progress toward goal attainment (e.g., 10,000 steps a day) [71], motivation came from recognizing that physical activity had the potential to take away pain or improve balance [9]. Older adults may also be more likely to track when asked by doctors, rather than for personal benefit [9].

When it comes to memory concerns, most research on tracking envisions people with cognitive impairment in more passive roles [84]. The research that does place people with memory concerns in more active roles typically focuses on supporting people in keeping track of routine tasks, particularly related to safety. This includes systems to support memory, such as through capturing interactions with everyday objects like stoves and doors to mitigate memory-related safety concerns [50]. Smart home technologies have been developed to offer older adults with memory concerns a method to track daily activities, such as remembering dates, tracking meals and medication, and enhancing independence and confidence by aiding in task recall and routine management [21]. One study that used a voice-controlled assistance system demonstrated improvements in older adults’ ability to recall scheduled activities such as taking medication [17]. Another study that designed a natural language interface used to ease medication management was able to reduce pressure to recall routines for older adults with mild cognitive impairment [57]. Researchers have also supported older adults with mild cognitive impairment by facilitating daily tasks and reducing anxieties related to memory lapses such as forgetting to turn off appliances [85]. These projects are meeting important unmet needs. However, some scholars have raised concerns that most research with older adults starts with a “solution to a supposed problem” (p. 3, [22]) rather than looking to what people want to [22] or are already [66] self-tracking. Our research responds by examining what older people with memory concerns are already self-tracking.

### 2.2 Emotion and Tracking

Self-tracking involves emotions in many ways. Self-tracking technologies may leverage positive and negative emotion in encouraging users to adopt healthier habits, which can be experienced as encouragement or shame [55]. For instance, one study analyzed the experiences of people using wearable activity trackers and discussed the role of positive and negative emotions in influencing user behavior [39]. Sometimes, the emotional effects are not intended in the design of the application, but are present due to the larger context of use, such as when individuals with eating disorders use weight loss apps [26] or when people struggling with fertility track menstrual symptoms [19]. Self-tracking also affects people’s social worlds. Some older adults have concerns about how monitoring technologies affect their interactions with others, such as reducing the time they spend with a health professional or caregiver [38].

Given social contexts and cultural meanings, any device involved in health and disability may bring up social emotions such as embarrassment, shame, or pride [72]. Wearables can be an “emotional technology” for older adults [80], and monitoring systems can give rise to emotional responses [9]. These emotions are often found to be negative. Self-tracking devices can involve the potential for stigma or anxiety due to a perceived similarity to a medical device [80]. Monitoring systems can be perceived as discouraging due to a focus on deficit and decline [9, 23]. There has also been much literature on how devices that track or monitor older adults’ well-being *for others*, such as wearable fall alert pendants, can be perceived as threatening independence and are therefore avoided (e.g., [9, 12]). Brewer offers alternatives to the standard approach of tracking and monitoring older adults’ negative health indicators such as falls or irregular heartbeats, noting that older participants preferred more “positive, strengths-based forms of health data” [8].

Negative sentiment around tracking may be heightened further when having to do with highly stigmatized cognitive impairment in older adulthood; Caldeira et al. discussed how in contrast to physical screenings, residents of a continuing care retirement community experienced anxiety when taking a yearly cognitive test [9]. Given how emotionally charged tracking can be for certain users, the goal of this paper is to take an analytic approach that helps surface unique and situated emotions that appear in our data.

## 3 Methods

Our procedures, participant characteristics, and analytic process are described below. All study procedures were approved by our University Institutional Review Board.

### 3.1 Procedures

Inclusion criteria for participation in the study were for participants to be at least 60 years of age and to self-identify as having memory concerns. We chose self-identification to enable us to sample data from people with a range of memory concerns, from uncertainty about whether they were experiencing changes to a medical diagnosis. It also might have helped us speak to people who would not have participated in a study that requires a diagnosis due to stigma or the challenges of obtaining a diagnosis [2, 67]. Therefore, in recruitment materials and interviews, we used the terms “memory concerns” and “concerns about your memory” rather than terms such as “cognitive impairment.”

We conducted 60-minute remote semi-structured interviews with participants who met the inclusion criteria. Participants first reviewed the consent forms and provided informed consent (there were no indications that any participants did not have the capacity to provide informed consent). Participants provided demographic information as well as information about the time they spent engaging in activities. We then asked questions about self-tracking, focusing on participants’ interest in, past experience of, and barriers related to self-tracking health data, both with and without technology. At different points in the interview, we brought up cognition or memory, explicitly (e.g., asking about a participant’s interest in writing things down to support memory) and more subtly. Over the rounds of recruitment, we adjusted our interview protocol to expand on areas that we found interesting as we reviewed our data, cover the topic more comprehensively through different angles, and find ways to talk about delicate topics (cognition in particular). The supplementary material includes the last iteration of the protocol.

### 3.2 Participants

We recruited participants from different avenues to achieve some diversity in terms of living arrangement, age, income, and race. Recruitment avenues included a group home for low-income older adults, a higher-end continuing care retirement community, and independently living individuals (clients of the same care manager in a program with specific inclusion criteria that included region, a particular religious minority group, and an income range considered to be low-middle income for that geographical area). Another round of recruitment sought to achieve more racial diversity in the study, and we continued to recruit across income ranges: from a university exercise program, as well as community-based organizations that serve low-income populations, who were asked to spread the recruitment material through their networks with a focus on recruiting people of color.

The majority of participants were located in the Midwestern United States. 29 participants went through study procedures. We stopped data collection at this point as we began hearing similar accounts and perspectives.

We asked participants to describe their memory concerns in an open-ended way when we collected demographics. Some identified minor concerns, and others reported a diagnosis such as Alzheimer’s Disease. Table 2 summarizes the ways that people (using pseudonyms) described their concerns about their memory.

In terms of health goals, most participants described physical activity (n=21) and weight management (n=15). Diet was brought up as distinct from weight management (n=10). Cognitive (n=6) and mental (n=8) wellness were brought up as distinct goals, with 5 participants bringing up a more holistic, general health goal. Sleep (n=3) and two unique goals were the least mentioned categories.

### 3.3 Writing Process and Style

We began to qualitatively analyze the data from interviews using the approaches we usually use when engaging in reflexive thematic analysis [7], which includes familiarizing ourselves with transcripts, memoing, and open coding, as well as discussions among the research team. However, we found that this approach did not capture the richness of the data that was obvious to us during interviews or discussions of the data. An example of an early memo was:

Some participants noted a link between physical health and mental health. Not only do they feel that physical health can boost their mood, but they feel that it can help their memory as well. Thus, they view exercise as a way to delay onset of memory problems.

Memos that began to note themes collapsed the tremendously varied experiences with self-tracking in our data. We decided that this could be best described through an alternative writing approach. We drew on anthropologist Kathleen Stewart’s affective writing approach, which involved generating an array of scenes based on real data. This approach, which has been used in past CSCW research to understand how techworkers were affected by the techlash [77], enabled us to keep the emotional richness that emerged during our interactions with participant accounts.

Before describing how we engaged in affective writing, we briefly describe how we understand “affect” in the vein of Stewart. Affect is not interchangeable with emotion [31]. Affects are more ambiguous and challenging to label. Affect is nonrational and nonconscious, becoming conscious only when it becomes a particular emotion. Though slippery, affect is important to study because it can tell us not only what is happening in a particular moment, but also helps us trace the conditions that make thoughts and feelings possible (p. 3, [73]). Unlike the thematic analysis approach we began with, affective approaches do not seek to paraphrase, categorize, or represent [75] data. Rather, they can show where affects “might go” and “what potential modes of knowing, relating, and attending to things are already somehow present in them in a state of potentiality and resonance” (p. 3, [73]).

Scenes were developed following procedures in Su et al. [77], and written and analyzed in several iterations. Scenes are based entirely on empirical data from participants. Scenes use participant language as much as possible, but are not excerpts from interviews as they are not first person – they are written in the third person, as they are created by the researcher. In addition, scenes can bring together statements made over the course of the interview. Finally, scenes can include researcher sentiment (identified as such in the writing).

The first author wrote a series of scenes based on individual interviews. Scenes were shared, along with the segments of the corresponding interview, with the second and last author of the research team. The team provided feedback and suggestions for each scene, including how effectively affect came through. Scenes underwent iteration, in particular, to directly use words, phrases, and speaking styles wherever possible. In total, we created 34 scenes.

To illustrate this process, we provide one example of this process of iteration with a scene that is included in this paper in its final form. The first author was drawn to create the scene after engaging with the following segments of a transcript in particular:

**P7:** One of the things I understand about memory is oftentimes, I will, people can, but I will remember the last memory of the event rather than the event. I’m trying to remember my 21st birthday. I don’t remember my 21st birthday anymore. I remember the last time I told somebody about my 21st birthday, that kind of thing. So I’m trying to figure out my memory when I come to a point where it’s completely wrong. You know, there’s still a full jar of popcorn kernels in the pantry. I know it’s there. I’ve seen it. And of course it isn’t there. What am I remembering? Why is the memory wrong? Because it’s so vivid that it was there. Those are the kinds of memory things that– I wouldn’t say they bother me. They intrigue me to the point of– But I notice I’m certain. The certainty is incorrect. So that’s what I would notice about memory right now. Being certain of something that is just not correct.

*Later in the interview when asked about self-tracking* P7: I will tell you this when I swim. I try to do laps. And I do try to get to 30 back and forth. And when I try to get to 30, I’m counting and I’m trying to count in, you know, the breaststroke and in the backstroke, and then if I get lost or confused, I do “Oh, well. It’s about 30”, and I’m okay with that. The idea of that tracking is very important that I try to get to my 30. And because we’re timed now in the pool in a half hour, can’t always get to 30 in that half hour. And just at that point of saying “Oh, you know, yeah, close enough”. I don’t have to write down for the next time I’m in the pool.

**First author:** And so does this sort of tie into the concerns that you have about your memory? This sort of mindset that it’s okay if you don’t remember exactly, you lose track of exactly what the data is, as long as you have a rough approximation.

**P7:** Yeah, yes. In that it’s not important. You know, I always try to tell myself that there’s only so much room inside [my brain] I had to cram stuff into it. I want to spend time focusing on remembering, Do I really have that popcorn in the pantry, then? Then I do that “it was 29 laps or 30”. And it’s not critical. “How important is it?” is a good mantra to try to catch on to.

The first author captured what they found most evocative in the following scene:

Who cares? P7 recognizes changes in his memory, such as forgetting that he has a full jar of popcorn kernels at home, or losing track of the number of laps swam when exercising, but he doesn’t care. Is it really that big of a deal? If he has to repeat an action, or sees that he’s misremembering something, what’s the issue? Memory changes and cognitive decline are just a part of life, and he’s accepted that. In fact, he’s more fascinated by it than anything. He takes the time to research it so he’s more informed about it.

The final version appears as the second scene in Section 4.1. To get to this final version, we met and identified changes that should be made to the scene to better and more accurately communicate affect. This included providing more space for the participant’s experience. In the initial scene, misremembering the popcorn kernels was listed quickly as an example of things the participant was forgetting, rather than an account of what this actually looked and felt like as experienced by the first author. In this sense, the first author’s own experience talking to the participant was key. In this particular scene, iterating in this way led to a final scene focused on Phillip’s work to cultivate a particular way of feeling in response to misremembering. Relating these changes to the initial scene back to Stewart’s writing style, the first scene was tidy, jumping quickly from the specifics (popcorn kernels and laps in the pool) to a description of Phillip’s outlook. Another way we sought to more accurately convey affect included returning to the transcript excerpts to ensure important-seeming words were language that participants used. For example, instead of describing Phillip as “fascinated” by memory changes, a term that he had not used, we edited the scene to use Phillip’s way of describing how he felt, which was “intrigued.” The revised scene is closer to the spirit of Stewart’s writing, which attempts to “slow the quick jump to representational thinking and evaluative critique long enough to find ways of approaching the complex and uncertain objects that fascinate...” (p. 4, [73]).

A paper that results from this kind of analytic approach looks different than a traditional qualitative paper with thematic subsections grouping common sentiments across participants. As Stewart argues, ordinary affects “are not the kind of analytic object that can be laid out on a single, static plane of analysis.” Scenes look different from each other, but involve “a tangle of potential connections” (pp. 3–4, [73]). To uncover and elucidate these connections, we went through a series of mapping exercises that involved arranging and rearranging scenes. Affectively writing about our data, and organizing and reorganizing scenes, led to our focus on potentials associated with self-tracking, the organization of scenes by what kinds of forces were most visible (individual, social, macro), and our research questions. As we conducted our analysis, we also became interested and drew out the ways people leave open or foreclose potentials given the ways that emotions appeared to be involved.

## 4 Findings

In the scenes below, readers will encounter tremendously varied accounts of self-tracking and associated potentials. Scenes are arranged by the structural forces that appear most clearly in each scene: at the individual, relational, and macro level. Each scene is accompanied by text that draws out how self-tracking appears (even when not referred to as such in participant words), potentials associated with self-tracking in that scene, and emotions that accompany these potentials. Scenes are separated by a fleuron.

### 4.1 Tracking on One’s Own

In the three accounts below, participants describe how they are, themselves, keeping track of different details in their lives. Some potentials are about whether things are as they are thought to be.

Marissa keeps tabs on what is happening outside by counting the birds and keeping track of other going-ons around her.

*Keep still so I can count you.* Marissa gushed about how much she loved the campus grounds. During her daily walks, she makes sure to stop by the aviary. Once there, she makes sure that all 15 birds are still there, though it is hard to count them because they move around so quickly.

When asked about her health-related goals, Marissa said she’d like to be able to continue moving. That’s why she walks. She does things to enhance her memory as well – or at least prevent total memory loss. This includes remembering the names of the hundreds of people that live around. She is competent at this. When she walks outside, she wants to see what things are blooming in the garden or if the fountains have been turned on yet, which, due to how early she takes her walks, she knows they haven’t yet. She finds it fun to encounter other people on her walk so she can say hello, greet them by name, and talk with them a bit. By doing this, she is bringing some joy to their lives.

Marissa keeps track of the birds, the names of the people that live around her, things that are blooming, and whether the fountains have turned on. This scene involves the potential, that she considers daily, of whether the 15 birds in the aviary are there, whether flowers are blooming, and whether the fountains have been turned on yet. There seems to be a comfort in narrowing this potential by, in the case of the birds, counting them as best as she can while they fly around. Reading her scene, it does not seem like Marissa will be displeased if she cannot entirely foreclose the possibility that the birds are not accounted for or whether the fountain is on or not – the routine of trying to do so is what is engaging.

Marissa also proudly brings up how even with memory concerns she is facing, she remembers the name of every person she meets. Here certainty does seem important.

Phillip also has daily routines involving tracking things that affect him. This includes that natural kind of tracking that many of us doing when we inventory our pantries and manage grocery shopping lists. He also self-tracks his activity toward a goal, specifically, toward a certain number of laps in the pool.

*Is it really that big of a deal?* Phillip has noticed vividly remembering things that are wrong. But he always tries to tell himself that there is only so much room inside his brain to cram information. So, when he is sure there is a jar full of popcorn kernels in his pantry, he knows it’s there, he’s seen it, and of course it isn’t there, he’s not bothered – he is intrigued. He recognizes changes in his memory, but he doesn’t care. It’s not critical. He says a good mantra to try to catch on to is: “How important is it?”

In the pool, he aims for 30 laps and tries to get there, but if he gets lost or confused while counting the number of laps he swam, he can tell himself, “Oh, well. It’s about 30.” He doesn’t need to write it down to come back to next time. And he’s okay with that. He recognizes changes in his memory, but he doesn’t care. Is it really that big of a deal? If he has to repeat an action, or sees that he’s misremembering something, what’s the issue? Memory changes and cognitive decline are just a part of life, and he’s accepted that. In fact, he’s more intrigued by it than anything. He takes the time to research it so he’s more informed about it.

Phillip keeps track in his head, even knowing the potential that getting things wrong is there. Through the comforting mantras he tells himself ("how important is it?"), he is cultivating the feeling of gentle curiosity as he considers this potential. But even while recognizing that errors in memory are not very important, Phillip does not discard his attempts to track or resolve potential – he still aims for a certain number of laps. He has accepted the cognitive changes that are a part of his life.

Elise feels differently about the memory changes she is noticing. These memory changes are affecting her ability to keep tracking her medication routine in her head.

*Not exactly denial*. Elise has been noticing memory changes in herself, but she is not willing to accept them just yet. She says that she probably should have some kind of marking system to remember to take her eye drops, but she also feels that she should be able to remember the eye drops because that’s her fallback pattern. She ***was*** able to remember at one point. She knows logically that her cognitive function isn’t as good as it once was, but she can’t bring herself to accept that emotionally. She’s aware that her current inaction is detrimental – she’s forgetting to take her medicine. But if she relies on external tools to support her memory, she’s resigning to a mindset that she’s not ready to accept.

Elise brought up a marking system as a form of tracking that would lead to a more reliable eye drop routine. However, self-tracking in this manner would mean realizing a possibility she is not willing to consider right now (that her memory has declined). She is not ignoring this potential. But she is keeping it ambiguous by not changing her method of tracking her medication at this point in time. Like in the previous two scenes, considering whether to keep potentials open or seek to close them involves feelings of discomfort or comfort, reassurance or disturbance.

### 4.2 Tracking with (and for) Others

In the scenes below, others are involved in the ways that participants keep track. The potentials here have to do with relational goals.

Bennett uses a number of tracking tools to stay accountable to the commitments he makes to others.

*Accountable to others to be accountable to himself.* Bennett, who had health-related goals including increasing socialization, used to be very into fitness, body-building, and all of that, but stopped 20 years ago. Now, he has been trying to reignite that motivation. He is trying to locate a person who works out at the same gym or at the same time. His difficulty finding someone is a major, major setback for him to begin weightlifting again.

Part of the reason Bennett wants a workout partner is to increase his motivation to workout and feel more responsible to keep up with his exercise. If Bennett is exercising by himself, he can let himself down. However, if he has made exercise arrangements with another person, then he’ll feel a sense of accountability to be responsible for following through with those plans.

This accountability is so important to him that he’ll be sure to set an alarm on his phone so that he won’t forget about engagements. These alarms are very crucial in helping him to remember the small things he has scheduled with others throughout the day. He gave the example of setting an alarm to show up for this study. He’s concerned that his memory lapses will affect his ability to follow through with plans and to be accountable to his commitments. What concerns him most about these lapses is that they will affect his integrity in being accountable.

Bennett is bringing in others to help him works towards the potential of getting back into fitness activities. However, the mechanism by which bringing in other serves him – bringing a sense of accountability – introduces a negative potential. Due to his memory lapses, he might forget commitments that he has made. He is minimizing this potential by setting alarms on his phone to keep track of engagements he has throughout the day. Doing so seems to help manage negative feelings around the potential of losing his integrity as he faces memory lapses.

Marissa has also looped in others, in face of the potential of looming memory changes. She is asking others to help keep track of her memory status, recognizing the potential for a lack of self-awareness.

*If you notice something, be sure and tell me.* Marissa’s husband had serious dementia towards the end of his life, to the point where he forgot how to eat. If there were TVs or computers around, he couldn’t figure out how to turn them on. Now that she’s seen this happen, she has some concern that this could happen to her. She’s told her kids, “If you notice something, be sure and tell me.” She uses tools to help manage her memory changes, such as setting out her equipment the night before and making lists, and she thinks this helps. She doesn’t think her memory changes are a concern now, but she realizes that she might not be aware of the things she is forgetting. She doesn’t want her memory changes to be a problem between her and her kids.

I (first author) was thinking this might be her reflecting on her experience of dealing with her husband’s condition and that she’s more worried about how her memory loss is going to affect the ones around her rather than how it will affect herself.

Potentials in Marissa’s scene include developing dementia – and also not knowing it. She develops a collaborative approach to track her memory changes, asking her kids to tell her if they *notice something*. She is doing this to avoid a second, relational potential of having memory changes that could “be a problem between her and her kids.” Bringing in others, like in Bennett’s scene, seems to help foreclose negative possibilities and thereby bring comfort.

Tessa, like Marissa, is tracking to work towards a better future for others.

*A better tomorrow?* When asked about her health-related goals, Tessa said that she wants to stay active. For her, this includes following politics and what’s going on in the world, and just trying to stay engaged in society. Keeping up with politics and news has always been important to her, but even more so now that she has grandchildren. She wants to see a good world for her granddaughters. She says she doesn’t think there is anything that she personally can do. However, in her next breath, she says she tries to stay aware so that she can fix some of the issues she is observing in the world – if she can. The thought that there is something she might be able to do is always in the back of her mind.

Tessa stays engaged in society, tracking the news and following politics. There is reassurance in tracking the news because this opens the potential of doing something about it. For Tessa, keeping this potential unresolved, rather than needing to commit to whether she can make a change that helps her granddaughters’ futures, seems helpful.

### 4.3 Tracking Influenced by the Macro

In the next set of scenes, we trace tracking and its associated potential to societal structures and systems. Weight goals and dementia management are structured by societal factors including medical institutions and cultural messaging.

Frequent self-weighing is a component of many weight loss interventions, with a body of evidence indicating a positive association between self-weighing and weight loss [83]. Terry has complicated feelings around tracking her weight with the scale and the potential of losing weight.

*It’s just a pipe dream.* When asked about her health-related goals, Terry immediately said weight. Terry has wanted to lose weight her entire life. She has always been on the heavier side, and she remembers having this goal of losing weight as early as grade school, when she daydreamed of having her legs thinner than the girl in front of her. Working nights for many years destroyed her eating and sleeping habits, and cooking for four boys didn’t help either.

Now, at 84, she says it looks like she’s never going to make it because that is just how her body works. She says it is more of a dream than a goal. She is in pretty good health (knock on wood) and says she realizes that the weight goal is more appearance-based than anything else. A breath later, though, she says that she can’t tolerate seeing an exact number for her weight. She tries not to use the scale. It’s hidden. She can tell how she is doing by the fit of her clothes and by how she feels.

Like in prior scenes, keeping potential suspended is useful. Doing so may allow her to keep working towards the possibility of weight loss without facing major disappointment if she does not succeed. Relatedly, the looser approach to tracking through the fit of her clothes may be more motivating than an immutable number on the scale.

While weight loss is an individual goal for Terry, gendered ideals and ways of living as well as national concerns around weight and health play a role.

Ian went to the doctor to address his memory loss.

*Does it even register?* Ian has had concerns with his short-term memory loss. The memory loss is not a massive drop-off, but it’s enough that he wanted to ask his doctor if he needed to go to a memory clinic. The doctor basically gave him a test for dementia – easy tasks like drawing a house or clock face. Laughing dryly, he remembers thinking, “Doctor, this is not really what I’m talking about.”

Still, one part of the exam did leave him feeling unsettled. He was asked to name nouns starting with a certain letter, and he didn’t do well on that. As someone whose job depends on quickness with language, this bothered him. His doctor said that there’s maybe something there but never followed up on what the issue could be. He didn’t feel taken seriously.

The doctor has given Ian a screener for dementia (likely the clock-drawing test [1]). Since he passed the screening, the doctor is not providing a referral (e.g., to a memory center) nor resources about dementia or information about possible causes of these changes (it could be the path to mild cognitive impairment or a type of dementia; it could be a medication side effect, other condition, or depression). Now, nobody really seems to be keeping track of his language abilities or short-term memory loss except him.

Ian is left unsettled; he has not left the doctor’s visit with any way to understand these observable changes in his cognitive abilities. He is left with the potential of having some memory impairment. Unlike some of the earlier scenes, it seems like Ian would prefer to foreclose the potential and figure out if something is going on.

Phoebe, unlike Ian, has made it into a system where her cognitive changes are regularly being monitored. Phoebe has registered the potential of getting better. This is not shared by her healthcare provider.

*Can it really not get any better?* Concerned about her memory, Phoebe purchased a book by Massachusetts General Hospital titled “Combating Memory Loss”. She hasn’t read the entire book, but she has read parts that are relevant to her. Her readings led her to believe that she could make lifestyle changes which would combat her memory loss. When she brought it up to her doctor, he didn’t seem to think that was the case. Instead, he simply administers memory tests, such as drawing a house or repeating after him, and tries to figure out how quickly her memory is changing or not changing.

It was strange to her to have her healthcare provider contradict what she had previously researched on her own, and that was sort of the end of the discussion. It’s disappointing that her doctor doesn’t give her anything to feel like there’s a chance of combating this memory loss. She doesn’t completely believe her doctor that nothing can be done because she has read from multiple outside sources that this impossibility of improvement isn’t the case.

The message from the US healthcare system is ambiguous. Massachusetts General Hospital says that lifestyle changes have the potential to combat memory loss. When Phoebe asks her doctor for assurance and guidance as to how this applies to her in the clinical encounter, the doctor not only does not assist but also says this is not the case. Phoebe is left on her own to deal with this potential.

## 5 Discussion

The first research question asked, *What potentials can be traced to the ways that older people with memory concerns are already engaging in self-tracking?* Scenes drew out potential, which we have defined as participant perceptions and emotions regarding what futures are possible for them. We traced these potentials to an array of forms of self-tracking including through technology, on paper, and in one’s head. We can group potentials associated with self-tracking as:

- having forgotten something (pantry items and number of laps for Phillip, commitments for Bennett)
- that something is in/not in place (birds for Marissa, pantry items for Phillip)
- having memory decline or cognitive impairment (Elise, Marissa, Ian)
- improving one’s cognitive state (Phoebe)
- losing weight (Terry)
- exercising more regularly (Bennett)
- damaging a relationship (Marissa)
- improving the world for others (Tessa)

Some of these potentials do not look that far removed from existing initiatives seeking to help older people with cognitive concerns keep track of things, detect cognitive impairment, and preserve relationships. Some introduce new insights, such as that people with cognitive concerns may have goals such as losing weight and exercising that can be supported with their active involvement, as well as that they may have higher-level goals such as improving the state of the world. However, grouping across participant accounts in this way obscures major differences between seemingly common potentials, such as very different attitudes towards the potential of having cognitive impairment.

We need to also bring to the forefront the emotions that come with these potentials to better understand the diversity of ways individuals respond to and employ self-tracking, which is answered in part by *RQ2: What emotions become visible as people consider these potentials?* Emotions in scenes included:

- engagement and intrigue (Marissa towards the birds, Phillip about remembering things wrong)
- positive anticipation mixed with aversion, concern, or disappointment (Terry’s dream of losing weight, *and* not being able to tolerate the scale; Phoebe’s idea of getting better cognitively and disappointment that she might not be able to; Bennett towards exercise and concern about not being accountable)
- unsettledness (Ian about the possibility of having some kind of cognitive impairment but not knowing it)
- concern (Marissa about having dementia and damaging her relationship with her children)
- resistance (Elise towards the potential of having cognitive impairment)
- acceptance (Phillip towards the potential of having cognitive impairment)

The third research question asked *What are some of the forces structuring tracking-related potentials for older people with memory concerns?* The forces we found to structure potentials included individual routines, relationships with others, and macro-level institutions and cultural messaging. In addition to answering the research questions above, our findings surfaced different emotions and motivations towards foreclosing or leaving open potentials.

While understanding the span of potentials, emotions, and forces in the scenes is a valuable starting point, further discussion is necessary to unpack the complexity of the unique relationships between them, which we turn to in the remainder of the discussion. First, though, we acknowledge that our findings do not easily translate into an understanding of how to design self-tracking tools that would work for all of the people in our sample, let alone broadly for people with concerns about their memory. This likely is due to our open-ended method and also because self-tracking was so naturally integrated into many different aspects of participants’ lives. Below, we seek to draw out what technology researchers can do with these findings, while acknowledging that there is merit to research that also parses what kinds of technologies *not* to design [5] based on these scenes.

### 5.1 Health Goals and Self-Tracking Practices are Purposeful and Varied

Existing tracking initiatives for people with cognitive concerns do not yet reflect the health goals and existing self-tracking practices that participants in our study cared about, prioritized, and acted on day to day. We describe these mismatches and offer accompanying design implications.

#### 5.1.1 Expanding our understanding of health goals.

The goals that existing tracking initiatives work towards, such as safety, early detection, and medication adherence support look very different than those in participant scenes. Existing initiatives share the biomedical focus of most research on age-related cognitive impairment [6, 18, 60]. This is not to say that medical, health, and memory-related concerns were not important to participants. Rather, participants framed goals in terms of being accountable to others or improving the state of the world as much as they did exercising and staying cognitively engaged.

We are not the first to argue that more expansive views of health are needed in the context of self-tracking with older adults. Researchers have noted that health goes beyond physical health for older adults [22, 64, 79, 82]. This has led to recognizing how a range of naturally occurring uses of self-tracking, such as keeping track of recipes, can be considered within the realm of health and active aging [64]. Some of the indicators tracked in our study go beyond this past work into realms that may seem unrelated to health and self-tracking. Two notable examples are Marissa keeping track of birds and Terry keeping track of the news. Should these be seen as within the realm of self-tracking towards health goals? We would argue that, for these participants, they should. Lupi and Posavec paved the way for this expansive view of self-tracking^1^ with their year-long project exchanging drawings of their personal data. The project begins with a “week of clocks,” where Lupi and Posavec track data, such as when they checked the time or the type of clock used, and visualized the data through drawings. They explain that this topic might seem “impersonal” but in fact facilitated their “tell(ing) each other the stories of their days through their data” (p. 2, [54]). Birds and news may similarly seem impersonal, not involving the *self* part of self-tracking. However, these “indicators” were tracked due to the meaning that they had. And, they were certainly related to health, with daily walks facilitating tracking birds, and tracking the news as a way of staying active.

An implication is to elicit personally meaningful and situated health-related activities from participants, rather than adhering to predetermined categories of what constitutes health-related self-tracking. How might a researcher in the domain of physical health elicit the motivations of Marissa’s daily walks, which include cognitive exercise, mindful engagement, social interaction, and bringing joy to others? Narrative inquiries involve eliciting stories along with their cultural, social, and institutional context [16]. This could be a promising approach for studies focusing on a particular health goal to gather situated experiences that are challenging to uncover through interviews or observations.

#### 5.1.2 Recognizing that people with memory concerns are doing work to take care of themselves.

Existing initiatives typically assume a very passive role for people with existing or potential cognitive impairment [84]. Our study demonstrates myriad ways that people with memory concerns are already self-tracking to support their well-being. This recognition connects our findings to self-care research, which points to the importance of surfacing and supporting “practical, routine, or banal” [62] activities for people with chronic health conditions. We see parallels to the importance of mundane care work for the people with memory concerns who participated in our research, though they do not necessarily require regular care or treatment like the subjects of self-care research in past work. Nunes and Fitzpatrick have called attention to how practical self-care work that people with Parkinson’s disease do often goes unrecognized by clinical and technology researchers [63]. Understanding that existing practices are legitimate (e.g., that people with Parkinson’s sometimes delay taking symptom-relieving medication so that they can use it before an important activity) [63] can help shift our approaches from trying to manage people in ways that may not be warranted (e.g., making sure people are taking their medications on time, without exception). Should we encourage people to take risks in addressing difficult and uncertain questions through self-tracking? An important line of research in personal informatics emphasizes self-experimentation [40], where individuals adjust certain conditions (e.g., trying a new medication), track their data, and analyze it to answer personal questions (e.g., Does the medication work for ME?). While some participants preferred to remain uncertain, individuals like Ian or Phoebe, who strived to resolve ambiguity and gain a deeper understanding of their memory concerns, might be willing to use a self-experimentation tool to explore personalized solutions – despite the inherent risk.

#### 5.1.3 Designing for individual, emotional relationships with self-tracking and health goals.

Existing tracking initiatives typically leave no room for the situated needs of an older person with cognitive concerns. For example, research on sensors to detect cognitive decline has rarely considered whether this is desired information.

HCI research has revealed that self-tracking can be emotional, often in negative ways, for older adults [9, 80]. Our study deepens our understanding of how self-tracking is emotional for some people with memory concerns by surfacing different kinds of emotions related to self-tracking – and providing evidence of how these emotions can shape decisions about self-tracking. Broadly speaking, self-tracking was associated with positive emotions in our study. Self-tracking seemed to play a supportive role as people aged and faced cognitive decline. However, negatively valenced emotions arose as well, and shaped decisions to track in one’s head versus a tool (paper or technology). Terry uses the fit of her clothes to gauge weight (keeping track in her head), because she cannot tolerate seeing a number on the scale (a tool that quantifies weight). Elise, for now, is tracking in her head, rather than using a marking tool, because she does not want to accept the potential of having cognitive impairment. Not all challenging emotions drive people away from tracking on paper or with technology. Bennett opts for general purpose technology rather than keeping track in his head, spurred by the desire to avoid missing a commitment due to a memory lapse.

Great care should be taken when selecting indicators and forms of tracking for sensitive topics, as these decisions may have significant emotional impact. For example, for participants with charged experiences around weight like Terry, a standard smart and connected scale would probably not be used. To make individually tailored approaches feasible, we can support people in identifying and self-tracking individualized measures through personalized tools [3, 44].

### 5.2 Ambiguous Potentials can be Desirable

The literature indicates that uncertain potential seems to be a feature of living with memory concerns in older age. Finding out whether memory changes are due to dementia has been described from the patient side as “confusing and labyrinth like” [67]. Clinically, attempts to resolve the nebulousness around what is “normal aging” versus pathological memory decline have gone on for decades [18]. What even constitutes having Alzheimer’s disease is up for debate (in its current form, whether biomarkers without symptoms warrant diagnosis [58]).

Existing tracking tools for people with memory concerns, then, seem to have no reason to intentionally leave room for ambiguity. It makes sense to design tools to definitively let someone know if a loved one with dementia is lost or if someone should seek clinical follow-up for possible dementia. In our findings, Ian and Marissa (in her second scene) would likely be positive towards tools that could help them foreclose the possibility of having cognitive decline. However, our study reveals cases where ambiguous potentials are acceptable, or even necessary. Continuing with the example of tools that can detect cognitive decline, it was important for Elise to maintain ambiguity in terms of whether she is facing memory changes or not. An intervention that would confirm this potential she is not ready to accept at this point in time could be devastating.

Rather than seeing ambiguity as a challenge to overcome, some HCI researchers have argued for embracing ambiguity [30, 70]. Scholars have noted that, similar to our findings, ambiguity is a part of everyday activities – and that designs should not necessarily inhibit this. For instance, designs can specify look and feel [34] while leaving their role open to interpretation [70].

Ambiguity has been conceived of as a design material in work creating self-tracking devices supporting people in interpreting tracking data [24, 35, 69, 76]. A challenge in this space is finding balance between overly open-ended and overly scaffolded data representations [69, 76]. With respect to our findings, we may similarly recognize that data ambiguity can be useful as a design strategy [68]. We return to considering tools that might or might not work for Terry to flesh out how this could translate into design. Terry dreams of the potential for weight loss, but a scale will foreclose that potential. Building on prior work that imagines a scale providing a range but never showing point estimates of current weight [41], this scene suggests that scales can be designed to portray the kinds of ambiguous potential that Terry craves, alluding to the dreams of her childhood and therefore the positive, however unlikely, chance of succeeding.

In a similar vein, ethnomethodological work examining the potential of (ubiquitous computing) technologies to facilitate routines in domestic life speak to the outwardly “unremarkable” character of such activities [78]. By virtue of their unremarkable-ness, routines are difficult for computing systems to recognize and interpret. Indeed, in many of the scenes, there are aspects that are difficult to capture regarding how people self-track. It can be done in one’s head as they swim laps or watch the news. As Tolmie et al. [78] note, caution must be exercised before jumping to augmenting everyday artifacts in the environment with computing to preserve the different mediums in which tracking is done. For instance, we may pause before trying to augment Phillip’s swimming routine for those 30 laps he counts as best as he can – the point isn’t the exact count but the routine of exercising not only his legs, but his memory, regardless of whether he may have misremembered.

### 5.3 Forces Structuring Self-Tracking Practices of Older Adults with Memory Concerns

This section largely focuses on findings related to the third research question, which asked what forces structure tracking-related potentials for older people with memory concerns. Stewart notes that potential, and the emotions that surround it, do not exist apart from material, social, and political forces [75]. The forces we identified in the findings included individual routines, relationships with others, and macro-level institutions and cultural contexts.

While self-tracking can be viewed as an individual activity, researchers are increasingly calling attention to the ways that it can be collaborative [13, 53] and situated in a broader sociocultural context [37, 53]. This is also the case in the context of cognitive impairment, particularly when there is a diagnosis: with researchers pointing out collaborative technology use and decision-making around technology in dementia and mild cognitive impairment [25, 36, 57] and the ways that social assumptions shape possibilities of life with dementia [49]. Similarly, self-tracking in our study is not just an individual keeping track of something for themselves alone, with even individual routines involving others. Self-tracking is also socially negotiated with others (Marissa asking her children to monitor her), done to be recognized in a certain way (Bennett as someone who does not forget engagements), and a way to remain connected to the world (Tessa’s news, Marissa’s walks). Tracking opens the door to relational possibilities, which allow people to preserve themselves in relation to those they value most, *particularly important* given the very real, amorphous anxieties that can swirl about when memory declines.

Cultural meanings shape emotion [31, 75]. It is key, then, to understand the sociopolitical state of affairs that likely shaped participant experiences. The group of people who are 65 and over now and in the coming decades are seen as a looming crisis for healthcare systems and economies [33, 61]. Researchers and public dialogue frame the potential associated with dementia as a “catastrophe awaiting modern societies” [86]. Options for care and lifestyle interventions have been devalued in the pursuit of a potential cure through medication [6, 18, 60]. An understanding of societal commitments and attitudes are necessary to contextualize many of the scenes, but in particular the medical encounters: being told there is no way to get better given the lack of any cure for dementia (Phoebe) or challenging the only two options being “normal aging” or “mild cognitive impairment or dementia” (Ian).

Negative societal attitudes towards dementia affect people with memory concerns. There is a measurable phenomenon named “Dementia Worry” [42], perhaps explaining why older adults can sometimes fear scores of routine cognitive tests more than physical tests [9]. While negative sentiment around cognitive impairment shapes decisions to track, it does not determine them. While Elise avoided the marking system for eye drops because it would realize the potential of having cognitive impairment, Ian was eager to understand whether they have cognitive decline as it may impact their job. And, we see the power that individuals can muster to resist damaging dominant discourses when Phillip describes the work he has done to cultivate an open attitude toward his memory loss.

We can improve our research and design by recognizing that “there is always the weight of the world in what can be hoped for and what must be feared” [75]. Tracking technologies might be beneficial for people with cognitive concerns at certain times. Other times, people may be unsettled by the potential of having a feared condition or in the process of coming to terms with a disease’s consequences [63]. We have a moral opportunity when designing, say, a sensor system that detects cognitive impairment – how (or whether, or when) the output of the system is delivered must be considered, and on an individual basis. Incorporating affective insights into our research and design process may lead to more useful and emotionally accessible technologies for dementia detection, memory support, and well-being.

## 6 Conclusion

The scenes we produced based on our data convey a variety of ways in which tracking takes place and a range of associated potential for older people with concerns about their memory. There was diversity in terms of whether something like tracking cognition was welcome or not at a particular point in time. There were also differences in whether people wanted to resolve potentials or leave them open. Individual routines, relationships with others, and macro-level institutions and cultural contexts shaped potentials and associated emotions. In the spirit of this affective mode of writing, we emphasize that our reflections are our own, and the reader may reach their own based on reading our scenes.

## Supplementary Material

Supplemental Material - Tracking and its Potential for Older Adults with Memory Concerns

## Figures and Tables

**Table 1:** Participant Demographics. Four participants did not respond to demographic questions.

Characteristic	Participant responses
Age	60 - 83. Average age 70.2 (StDev = 8.2)
Race	White (n=14) Black or African American (n=6) Asian (n=3) More than one race (n=2)
Gender	11 male; 14 female (to the open-ended question “what is your gender?”)
Education	Master’s degree or higher (n=12) Bachelor’s degree (n=6) Associate degree (n=1) Attended some college (n=3) High school degree or less (n=3)
Comfort with technology	Very confident (n=6) Somewhat confident (n=12) Only a little confident (n=4)

**Table 2:** Participant ages and self-reported memory concerns

Pseudonym	Age Range	Concerns about Memory	Number of Years	Confidence Using Technology

Derek	80s	Forget names or words	Unsure	Somewhat
Julian	70s		5	Somewhat
Tessa	70s		2	Only a little
Clara	80s		30	Only a little
Brianna	70s		1	Somewhat
Phoebe	80s		7+	Unsure
Phillip	70s		Unsure	Mixed somewhat to only a little

Vivian	60s	Do not want to run into trouble later on with memory issues	“A few” years	Only a little
	
Marissa	80s		3	Somewhat

Nora	60s	Short term memory loss while doing a task and/or difficulty multitasking	2	Very
Ethan	60s		1	Very
Ian	60s		6–7	Somewhat
Malcolm	60s		2	Very
Lydia	60s		5	Very
Vincent	60s		8–10	Somewhat

Lana	60s	Not able to access longer-term memories	10	Somewhat
	
Joyce	60s		“Always ”	Somewhat

Giselle	60s	More general descriptions of memory changes	2	Very
Grant	60s		“Past few”	Only a little
Bennett	60s		5–10	Somewhat

Marcus	60s	Can’t always recall events (meals) from the previous day	6 months	Very

Simone	60s	Sometimes forgets to schedule things (rides)	3	Not disclosed

Naomi	70s	Forgets things a lot (e.g., where to put fingers to type)	4–5 years	Somewhat

Connor	80s	Not disclosed	Not disclosed	Somewhat
Sam	Not disclosed		Not disclosed	Not disclosed
	
Robin	Not disclosed		Not disclosed	Not disclosed
Dana	Not disclosed		Not disclosed	Not disclosed
	
Terry	Not disclosed		Not disclosed	Not disclosed
Elise	70s		3–5	Somewhat confident

## Footnotes

1 Lupi and Posavec describe their work as “personal documentary” and specifically not as quantified-self. They explain that this is because they are not concerned with efficiency (arguably an epistemological foundation of the quantified self-movement) [43]. Given our use of the concept of self-tracking does not include efficiency or even self-improvement, we believe our argument is a fair characterization.
