# Supplementary material for: Tracking and its Potential for Older Adults with Memory Concerns
Source: Proc SIGCHI Conf Hum Factor Comput Syst. Author manuscript; Available in PMC 2025 Jun 25. (PMC12188979; doi:10.1145/3706598.3714093)
Supplement: Supplemental Material - Tracking and its Potential for Older Adults with Memory Concerns [file NIHMS2078319-supplement-Supplemental_Material_-_Tracking_and_its_Potential_for_Older_Adults_with_Memory_Concerns.pdf]

Below are the questions that we will be asking during the interview. There is no need to have formulated answers going into the interview. We are just providing this in case you are curious about the questions or would like to reflect on them in advance.

There are many questions in this document, but we will not necessarily ask all of them--we will be mindful of your time and the topics you are most interested in!

The interview is "semi-structured," which means we can follow up with additional questions based on your answers. You can always choose to not answer any questions.

Interview Questions

PART 1: In this section, we will ask you about your health-related goals, and focus on exercise in particular.

1. Can you tell me about your health-related goals?
    1. *For each health-related goal, ask:*
        1. Why is this important to you?
        2. Do you experience any barriers when trying to achieve this goal?
        3. Do you feel like your memory concerns have an affect on the way you try to achieve this goal?
    2. One common health related goal is exercise. You mentioned this in yours/is this a goal for you?
        1. How much exercise do you try to get each week?
        2. What do you do for exercise?
        3. What are barriers that you experience to exercising?
    3. There are different ways of getting the health outcomes of exercise. There are intentional periods of exercise like we talked about but also, just trying to get around and move around after sitting for some time. So for example getting around each hour of watching TV.
        1. Would you say this is a goal you have?
        2. Can you tell me about a recent time that you tried to move around after sitting for a long time? [if they can't think of anything - then we get to the general question "How do you try to move around after sitting for some time?"
        3. Did you meet any barriers trying to do this?

4. Do you experience any barriers when trying to meet these goals?

PART 2: In this section, we will talk about self-tracking and ask if this is something you currently do or have ever done.

We are interested in learning about your health-related goals and if self-tracking can be used to keep track of and achieve these goals.

Self-tracking is a way for us to see things that we have done, are currently doing, or plan to do in the future. For example, a doctor may ask you to keep track of your blood pressure every day and send the data back to them. Another example would be keeping a journal to keep track of your mood or mental wellness.

However, you do not have to write down and record data for it to be considered self-tracking. If you were to check your watch when exercising to see how long you have been working out, that would be considered self-tracking. Another example is if you were to weigh yourself every morning to keep an eye on your weight.

1. Now that we've walked through tracking in detail, I want to ask if you have ever tracked any health related activities.
    1. What type of health information did you track? (you can list a couple, and then we can go in-depth one by one)
    2. How did you track the data? (e.g., a particular device? Recording on paper?)
    3. What did you do with the information that you recorded?
    4. Can you think of a particular time that tracking this information changed a daily habit/behavior?
    5. Is this account typical of how you used the information or was this a rather exceptional circumstance?
    6. *If method was with using technology:*
        1. Is there any way this can work or could have worked better for you?
    7. If you do not self-track data anymore, why did you stop?
        1. Would you be open to self-tracking health data again?
        2. Have there been any changes you've noticed in your own tracking over time? Changes like the frequency of tracking, or the types of things you track?
        3. Do you feel like your memory concerns affect the way you track this health information in any way?

2. *Has anyone else ever asked you to track anything related to your health? [if they say no: say for example, some people have had to track their blood pressure for their Dr. Ask the questions up top]*

3. Are there any other kinds of health-related information that you would like to track *if there were no barriers to doing so?*

4. *For a health related goal they mentioned at the beginning but not with tracking, ask:*
    1. *do we say- you mentioned health related goals of X (bbut didnt mention tracking). What are some of the steps you take towards this goal? How do you keep track of how you are working towards it (if at all)? How do you keep track of whether you met the goal (if at all)? How do you keep track of your progress over the long term (if at all)?*
    2. *If they don't, why don't you?*

PART 3: In this section, we will ask you more about self-tracking, but specifically about physical activity.

1. Now we will shift to discussing tracking *physical* activity. Would you be interested in tracking your physical activity data? And by this I mean number of times exercising, or number of steps, or even how much you moved around the house that day.
    1. *If yes:*
        1. So I mentioned the number of times exercising, or number of steps, or how much you moved around the house as examples of TYPES of data you could keep track of. What type of data would you want to keep track of?
        2. What do you think you might want to do with this information?
        3. How often do you think you might want to look at this information?
        4. There are a range of ways people keep track of their physical activity. This includes wearable pedometers that automatically track for you or even devices that you don't need to wear that detect motion. Do you see yourself drawn to one or the other? Why?
        5. Some people wear pedometers and note their steps with paper and pencil, while others might wear trackers that automatically upload their steps into a smartphone app. Do you see yourself drawn to one or the other? Why?

6. Are there any reservations you have about self-tracking data?
7. Do you have any idea of how your memory concerns might affect your use? For example, would this affect your frequency of use? The ease of use?

2. If not:

1. Why not?
2. Does your answer apply to other kinds of technology?
3. Do you see any value in self-tracking physical health data?
4. Can you imagine any barriers to using technology for self tracking physical health data?
5. Can you imagine yourself trying self-tracking physical health data under any circumstances?

PART 4: In this section, we will ask you more about self-tracking, but specifically about mental and cognitive health.

2. Now we will shift to discussing tracking mental activity. Would you be interested in tracking your mental health data? And by this I mean keeping track of your mood, writing things down to help support your memory, or anything else relating to promoting good mental or cognitive health?

1. If yes:

1. What type of data would you want to keep track of?
2. What do you think you might want to do with this information?
3. How often do you think you might want to look at this information?
4. Are there any reservations you have about self-tracking mental health data?
5. Do you have any idea of how your memory concerns might affect your use? For example, would this affect your frequency of use? The ease of use?

2. If not:

1. Why not?
2. Does your answer apply to other kinds of technology?
3. Do you see any value in self-tracking mental health data?

4. Can you imagine any barriers to using technology for self-tracking mental health?
5. Can you imagine yourself trying self-tracking under any circumstances?

PART 5: In this section, we will ask you more about technology and self-tracking if you mention that you use a lot of technology.

1. Would you be interested in adapting a tracking app to work best for your activities and movements?
   1. *If yes:*
      1. What activities and movements would you expect to be more difficult for an app to automatically track? Why?
   2. *If no:*
      2. Why not?

Now that we have come to the end of the interview, I'd love to hear your takeaway of what self-tracking is? What purposes do you think it might be useful for?

That brings me to the end of my questions. Is there anything else you would like me to know about anything we have discussed today?
